# TMS-EEG signatures of glutamatergic neurotransmission in human cortex

**DOI:** 10.1038/s41598-021-87533-z

**Published:** 2021-04-14

**Authors:** Paolo Belardinelli, Franca König, Chen Liang, Isabella Premoli, Debora Desideri, Florian Müller-Dahlhaus, Pedro Caldana Gordon, Carl Zipser, Christoph Zrenner, Ulf Ziemann

**Affiliations:** 1 Department of Neurology and Stroke, University of Tübingen, Hoppe-Seyler-Str. 3, 72076 Tübingen, Germany; 2 Hertie Institute for Clinical Brain Research, University of Tübingen, Tübingen, Germany; 3 CIMeC, Center for Mind/Brain Sciences, University of Trento, Trento, Italy; 4 Department of Basic and Clinical Neuroscience, Institute of Psychiatry, Psychology and Neuroscience, King’s College London, London, UK; 5 Department of Psychiatry and Psychotherapy, Johannes Gutenberg University Medical Center Mainz, Mainz, Germany; 6 Department of Neurology and Neurophysiology, University of Zurich, Balgrist University Hospital, Zürich, Switzerland

**Keywords:** Neuroscience, Neurophysiology

## Abstract

Neuronal activity in the brain reflects an excitation–inhibition balance that is regulated predominantly by glutamatergic and GABAergic neurotransmission, and often disturbed in neuropsychiatric disorders. Here, we tested the effects of a single oral dose of two anti-glutamatergic drugs (dextromethorphan, an NMDA receptor antagonist; perampanel, an AMPA receptor antagonist) and an L-type voltage-gated calcium channel blocker (nimodipine) on transcranial magnetic stimulation (TMS)-evoked electroencephalographic (EEG) potentials (TEPs) and TMS-induced oscillations (TIOs) in 16 healthy adults in a pseudorandomized, double-blinded, placebo-controlled crossover design. Single-pulse TMS was delivered to the hand area of left primary motor cortex. Dextromethorphan increased the amplitude of the N45 TEP, while it had no effect on TIOs. Perampanel reduced the amplitude of the P60 TEP in the non-stimulated hemisphere, and increased TIOs in the beta-frequency band in the stimulated sensorimotor cortex, and in the alpha-frequency band in midline parietal channels. Nimodipine and placebo had no effect on TEPs and TIOs. The TEP results extend previous pharmaco-TMS-EEG studies by demonstrating that the N45 is regulated by a balance of GABAAergic inhibition and NMDA receptor-mediated glutamatergic excitation. In contrast, AMPA receptor-mediated glutamatergic neurotransmission contributes to propagated activity reflected in the P60 potential and midline parietal induced oscillations. This pharmacological characterization of TMS-EEG responses will be informative for interpreting TMS-EEG abnormalities in neuropsychiatric disorders with pathological excitation–inhibition balance.

## Introduction

Transcranial magnetic stimulation applied during electroencephalographic recording (TMS-EEG) is a powerful technique to access excitability of the targeted brain area and effective connectivity to distant sites in healthy and in pathological conditions^1^. TMS-EEG is also emerging as an effective tool to examine impaired inhibitory and excitatory neurotransmission underlying a broad variety of brain disorders, such as epilepsy, schizophrenia, or Alzheimer’s disease^2–5^. To fully exploit clinical translation of TMS-EEG requires a solid physiological characterization of TMS-EEG responses, e.g., in pharmaco-TMS-EEG experiments using drugs with specific modes of action in the central nervous system.

EEG responses to TMS can be interrogated in the time^6^ and time–frequency domains^7^, providing complementary information about cortical processes^8^. The responses in the time domain are referred to as TMS-evoked EEG potentials (TEPs), which consist of a reliable alternating sequence of positive (P) and negative (N) peaks at approximately 30 (P30), 45 (N45), 60 (P60), 100 (N100) and 180 (P180) milliseconds after the TMS pulse when targeting the primary motor cortex (M1)^6^*.* Time–frequency decomposition reveals TMS-induced oscillations (TIOs) which, in contrast to TEPs, display responses not time-locked to the TMS pulse^9^. Their typical profile following M1 stimulation is characterized by an increase in the 8–30 Hz range in the first 200 ms, followed by alpha and beta desynchronization in the time window from 200 to 400 ms^10^, and a subsequent late beta rebound after 400 ms^11^.

Pharmacological studies in healthy subjects showed that the N45 and N100 TEP components are associated with GABA-A and GABA-B receptor-mediated inhibition, respectively. As an example, positive allosteric modulators of the GABA-A receptor, such as benzodiazepines and zolpidem increased the N45 TEP amplitude^12,13^, while the experimental compound S44819, a specific antagonist at the alpha-5 subtype of the GABA-A receptor, decreased the N45 TEP amplitude^14^. In contrast, the specific GABA-B receptor agonist baclofen increased the N100 TEP amplitude^12,13^. Moreover, these GABAergic drugs had a variety of effects on TIOs, indicating that TMS-induced power changes involve GABAergic inhibitory mechanisms^15^. Recently, a novel computational approach, termed PARAFAC has enabled the analysis of high-dimensional datasets, such as TIOs, to reveal low-dimensional descriptions of drug effects^16^. The multi-dimensionality of the TMS-EEG induced response profile, typically indexed over space, time, frequency, subjects and drug conditions, does not guarantee a straightforward extraction of specific drug-related effects. Recently, a novel computational approach, termed PARAFAC has enabled the analysis of high-dimensional datasets, such as TIOs, to reveal low-dimensional descriptions of drug effects^16^. The pipeline based on PARAFAC tensor decomposition allows a parsimonious description of the main profiles underlying the multidimensional data. Besides application in a wide range of EEG studies^17^, this purely data-driven approach shows utility in disentangling the effects of antiepileptic drugs on TMS-induced oscillations^16^.

Drugs acting on the excitatory glutamatergic system have so far not been tested with TMS-EEG measures, although glutamatergic neurotransmission plays a fundamental role in the excitation–inhibition balance to regulate neuronal excitability in cerebral cortex^18^. Moreover, the pathophysiology of many neurological and psychiatric conditions is linked to a dysfunction in the glutamatergic system, such as schizophrenia^19^, epilepsy^20^ or amyotrophic lateral sclerosis^21^.

Here we investigate the effects of two anti-glutamatergic drugs (dextromethorphan, perampanel) and the L-type voltage-gated calcium channel (L-VGCC) blocker nimodipine^22^ on TEPs and TIOs (analyzed both in the canonical way and with PARAFAC) in healthy subjects. Perampanel is a selective, non-competitive postsynaptic α-amino-3-hydroxy-5-methyl-4-isoxazole propionic acid (AMPA) receptor antagonist^23^, while dextromethorphan is a prodrug whose active metabolite, dextrorphan, acts as a non-competitive *N*-methyl-d-aspartate (NMDA) receptor antagonist^24^. AMPA and NMDA receptors are the main ionotropic receptors for glutamate in the central nervous system. AMPA receptor-mediated currents generate fast excitatory postsynaptic potentials (EPSPs), while NMDA receptor activation results in a prolonged EPSP that can last up to several hundreds of milliseconds. Action potential generation is largely controlled by AMPA receptor de/activation, while the slower kinetics of NMDA receptors enable spatial and temporal summation of postsynaptic potentials^25^. Accordingly, perampanel is used as an antiepileptic drug^26^, while dextromethorphan has demonstrated efficacy in reducing synaptic plasticity in human cortex^27^. Finally, L-VGCCs are not significantly involved in controlling the release of glutamate from presynaptic nerve terminals^28^ but block synaptic plasticity in human cortex^29^, probably through inhibition of calcium flux into depolarized postsynaptic cells^30^.

We expect that the anti-glutamatergic drugs will have significant and specific effects on TEPs and TIOs that will signify that AMPA and NMDA receptor-mediated neurotransmission can be tested with TMS-EEG responses.

## Material and methods

### Participants

Eighteen male participants (mean age ± SD: 26.0 ± 3.5 years, range 22–36 years), were included in this study. All subjects underwent physical examination, and were screened for possible contraindications to TMS^31^ and to the study medications. Inclusion criteria comprised of right-handedness laterality score > 70% (mean laterality score ± SD: 88 ± 15%) according to the Edinburgh Inventory^32^ and male gender, to avoid possible effects of the menstrual cycle on cortical excitability^33^. Exclusion criteria included presence or history of neurologic and psychiatric disease, use of illicit or recreational drugs, smoking, and a history of low blood pressure. The study was approved by the Ethics Committee of the Medical Faculty of the University of Tübingen (registration number 526/2014BO1), all methods were performed in accordance with the relevant guidelines and regulations, and all subjects provided written informed consent prior to study participation.

Sixteen subjects completed all experimental sessions. One participant did not finish the study due to medical conditions unrelated to the study and one other subject dropped out during the measurements. Therefore, all data analyses are based on 16 subjects.

### Experimental design

A combined pharmaco-TMS-EEG approach^12,14^ with a pseudorandomized, double-blinded, placebo-controlled crossover study design was employed to test the acute effects of single oral doses of perampanel, dextromethorphan and nimodipine on TEP amplitudes and TIOs.

During each experimental session, neurophysiological assessments were performed immediately before drug intake, and 2 h later, i.e., post-drug intake. Before each TMS-EEG measurement, resting motor threshold (RMT), defined as the minimum intensity sufficient to elicit a motor evoked potential (MEP) amplitude ≥ 50 μV in at least five out of ten trials was determined, according to the relative frequency method^34^. Then, resting-state EEG (3 min eyes open) was recorded, followed by the delivery of 150 monophasic single TMS pulses with a random interstimulus interval of 5 ± 1 s for TEP and TIO recordings. Due to different pharmacokinetics (Supplementary Table 1 in Supplementary Material), drugs and/or placebo were applied at two different time points between pre-drug and post-drug measurements (Supplementary Table 2 in Supplementary Material) to ensure serum peak concentrations during the post-drug measurements (Supplementary Fig. S1 in Supplementary Material). Participants received a single oral dose of perampanel (12 mg or 6 mg, Fycompa, Eisai Pharma), dextromethorphan (120 mg, Hustenstiller-ratiopharm Dextromethorphan, ratiopharm GmbH), nimodipine (30 mg, Nimodipin-Hexal, Hexal AG), or placebo (P-Tabletten Lichtenstein; Placebo capsules, Pharmacy of Tübingen University). All drug dosages administered in this study are approved for medical use. The order of drugs was pseudorandomized and balanced across subjects. To avoid carryover drug effects, consecutive sessions in a given participant were separated by at least 2 weeks.

### TMS-EEG data recordings

Participants were seated in a comfortable reclining chair throughout the measurements. They were instructed to keep their eyes open and to fixate a small black cross in front of them to minimize eye movements. Their right hand and arm were comfortably placed and kept voluntarily relaxed throughout the experiment.

A TMS-compatible EEG amplifier (BrainAmp DC, BrainProducts GmbH, Munich, Germany) with 62 high-density TMS-compatible C-ring slit EEG electrodes (EASYCAP, Germany) arranged in the International 10–20 montage were used to acquire EEG, hardware-filtered between 0.016 and 1000 Hz and digitized with a sampling rate of 5 kHz. To monitor eye movement and blinking, two additional electrodes where placed above the right eye and at its outer canthus. All electrode impedances were maintained at < 5 kΩ throughout the session. In order to avoid possible EEG contamination by auditory evoked potentials caused by the TMS coil discharge click^35^, white noise was delivered to the participants through earphones during the TMS-EEG recordings^36^. The sound pressure level was calibrated until participants indicated that they could no longer hear the TMS clicks.

TMS pulses were applied to the hand knob of the left M1 using a focal figure-of-eight coil (external loop diameter: 90 mm). The coil was connected through a BiStim module with a Magstim 200^2^ magnetic stimulator (all devices from Magstim Co, Whitland, Dyfed, UK) with a monophasic current waveform. The coil was oriented with the handle pointing backwards and 45° away from the midline, to induce current in the brain oriented from lateral-posterior to anterior-medial^37^. The optimal coil position to elicit MEPs in the right abductor pollicis brevis (APB) muscle was determined as the site that produced consistently the largest MEPs using a stimulation intensity slightly above RMT (motor “hotspot”)^34^. MEPs were recorded through surface EMG electrodes (Ag–AgCl cup electrodes) in a belly-tendon montage. The EMG signal was recorded using the Spike2 software (Cambridge Electronic Design). The EMG raw signal was amplified (Digitimer D360 8-channel amplifier), bandpass filtered (20 Hz–2 kHz) and digitized at an A/D rate of 10 kHz (CED Micro 1401; Cambridge Electronic Design). For constant coil placement throughout the experiment, the coil position at the APB hotspot was marked on the EEG cap. All TMS pulses were applied to the APB hotspot at an intensity of 100% RMT^12,14^, to limit possible contamination of TEPs and TIOs by re-afferent signals from MEPs^10^. The RMT was re-tested at the beginning of the post-drug measurements (Supplementary Fig. S1 in Supplementary Material) and, if different from pre-drug RMT, TMS intensity was adjusted to keep effective activation of the left M1 constant across pre- and post-drug measurements.

### Data processing

EEG data processing and analysis were performed using customized analysis scripts on MATLAB R2016a and the Fieldtrip open source MATLAB toolbox^38^. The continuous EEG data was segmented into epochs from − 600 to 600 ms relative to the TMS pulse. EEG data from 1 ms before to 15 ms after the TMS pulse contained the TMS artifact and were removed and spline interpolated^39^. Afterwards, data was down-sampled to 1 kHz. Bad trials and noisy channels were removed by means of visual inspection of the EEG epochs [percentage of removed epochs (mean ± SD): 25.4 ± 12.0%; number of removed channels (mean ± SD): 4.5 ± 2.5]. Then, independent component analysis (ICA) was applied to the EEG data in a two-steps procedure^40^. In a first ICA step, TMS-related artefacts were removed [number of removed components (mean ± SD): 4.3 ± 2.6]. Subsequently, the data was filtered with a 1–80 Hz 3rd order Butterworth zero-phase bandpass filter and a 49–51 Hz notch filter. ICA was then performed again and components representing physiological (i.e., eye blinking or eye movements, muscle artifacts), electrical or TMS related artefacts were removed [number of removed components (mean ± SD): 13.6 ± 6.2]. Successively, removed channels were interpolated using the signal of the neighboring channels^41^ and data were then re-referenced to linked mastoids (average signal of EEG electrodes TP9 and TP10). Finally, data were baseline-corrected by subtracting the average of the signal in the time window from 600 to 100 ms prior to the TMS pulse^12^ and smoothed with a 45 Hz 3rd order Butterworth zero-phase low-pass filter. TEPs were analyzed channel-wise, by averaging the EEG data of all retained trials, separately for the pre- and post-drug measurements.

For MEP analysis, EMG data were epoched from − 100 to 100 ms around the TMS pulse. An epoch was discarded if the absolute value of the average EMG signal − 100 to 0 ms before the TMS pulse exceeded a pre-innervation threshold > 0.02 mV. The percentage of discarded epochs due to pre-innervation was (mean ± SD) 11.0 ± 17.9%.

### TMS-induced EEG oscillations (TIOs)

TIOs represent changes in spontaneous oscillatory activity that are not time-locked to the stimulus onset^42^. The TMS-induced activity in the time-domain was isolated by channel-wise subtracting the average evoked response from each single trial^15^. Subsequently, time–frequency representations (TFRs) of the obtained data were calculated by convolving single trials with complex Morlet wavelets in the frequency range from 6 to 30 Hz in steps of 1 Hz and shifting the center of the wavelet in steps of 10 ms in the time window – 600 to 600 ms relative to the TMS pulse. The length of the wavelet was linearly increased from 2.5 cycles at 6 Hz to 7.5 cycles at 45 Hz. TFRs of power were obtained by taking the squared absolute values of the complex time series resulting from the wavelet transformation. They were then trial-wise z-transformed based on the mean and standard deviation of the full-length trial as described in Ref.^15^ and baseline-corrected by subtracting the mean value of the baseline period (from 600 to 100 ms before TMS), to ensure that the average pre-TMS values did not differ from zero and that z-values could be interpreted as modulation of the pre-TMS oscillatory activity. Finally, TFRs were averaged over all retained epochs separately for the pre- and post-drug measurements, and trimmed to remove the time points where no time–frequency values could be calculated (from − 600 to − 400 ms and from 400 to 600 ms with respect to the TMS pulse, corresponding to 1.25 cycles of a 6 Hz oscillation, the lowest frequency analyzed). Based on previous literature^12,16^, TIOs were initially analyzed in four a priori defined time–frequency regions of interest, enclosing the early (30–200 ms) and late (200–400 ms) responses in the alpha (8–12 Hz) and beta (13–30 Hz) frequency bands (Supplementary Fig. S3 upper panel, Supplementary Material).

### PARAFAC applied to TIOs

To further investigate possible drug effects on TIOs, a tensor decomposition analysis was also applied to the same preprocessed datasets. For the whole TFR window of interest (8–30 Hz with 1 Hz frequency resolution, 30–400 ms after the TMS pulse with 10 ms temporal resolution) without a priori assumptions, an array of 62 × 23 × 38 elements (62 channels, 23 frequency steps, and 38 time samples) was considered for each participant. Then, a 5D tensor was constructed, consisting of the three individual dimensions of topographical space, frequency and time, plus two further dimensions consisting of subjects (n = 16) and conditions [8: 4 drugs × 2 times (i.e., pre-drug, post-drug)], in order to account for all possible interactions with the effects of drugs on the subjects. Such 5D data array can be effectively approximated via a sum of *N* rank-one tensors, which represent the principal components underlying the TFR^16^.

In our case, the number of tensor components optimally representing the data was chosen to be four for two reasons: first, the set with four components was clearly encompassing one different (regarding topography, time and frequency spectra) component more than the set with three. This was not the case with the five components tensor set with respect to four, with two components becoming redundant on different dimensions. Secondly, it has been shown in an analogous analysis on the effects of antiepileptic drugs on TIOs that *N* = 4 components is the highest number where the explained data variance by the considered components reaches a plateau^16^. The effects of drugs on the subjects were then tested by contrasting conditions. For clarity, the non-negativity constraint was applied to all dimensions during the decomposition. Therefore, each array element in the decomposed tensors is larger than or equal to zero.

### Statistics

#### Resting motor threshold (RMT) and motor evoked potential (MEP) amplitude

A repeated measure analysis of variance (rmANOVA) with the within-subject effects of DRUG (4 levels: perampanel, dextromethorphan, nimodipine, placebo) and TIME (2 levels: pre-drug, post-drug) was run with MATLAB R2016a on the RMT and MEP amplitude data. The Shapiro–Wilk test was applied to test for normal distribution. The MEP data were log-transformed to achieve normal distribution. Sphericity was checked using Mauchly’s test and, whenever violated, the Greenhouse–Geisser correction of the degrees of freedom was applied. For all tests, the significance level was set to p < 0.05.

#### TMS-evoked EEG potentials (TEPs)

Five non-overlapping time windows of interest (TOIs) were a priori defined based on the group average TEPs of all subjects, pre- and post-drug measurements, four drug conditions and all EEG channels. TOIs were centered around the latencies of the canonical M1 TEP peaks P30, N45, P60, N100 and P180^12,43^. Specifically, TOIs were set at 16–34 ms (P30), 38–55 ms (N45), 56–82 ms (P60), 89–133 ms (N100), and 173–262 ms (P180) after the TMS pulse. As a first step, drug-induced TEP modulations were evaluated for each condition and TOI using channel-wise paired-sample t-tests. To address the multiple comparison problem due to the large number of tests (channels and time bins), a cluster-based permutation approach was used in a non-parametric framework^44^, as implemented in Fieldtrip (http://fieldtrip.fcdonders.nl/). This approach tests the null hypothesis that data in the experimental conditions are drawn from the same probability distribution. Significant clusters showed *t*-values resulting from the paired-sample *t*-tests exceeding an a priori defined threshold of p < 0.05, based on neighboring channels and time points. The minimum number of channels below the significance threshold to form a cluster was 2. The *t*-statistics at cluster level was computed in a second step by summing the *t*-values within each cluster and taking the largest of the cluster level statistics. A reference distribution of the maximum of the summed cluster *t*-values was obtained by re-applying the same procedure on the data randomized across the pre-drug vs. post-drug measurements. 1500 randomizations were used to obtain the reference distribution and the null hypothesis rejected if less than 5% of the permutations used to construct the reference distribution yielded a maximum cluster-level *t*-value larger than the one observed in the original data. Since the TEPs in the different TOIs are not independent observations but rather correlated to some degree, a False Discovery Rate (FDR) approach encompassing positively correlated events^45^ was used to correct for multiple cluster occurrence with the 5 TOIs. The same cluster-based approach was used to assess differences between TEPs in the pre-drug measurements of the four DRUG conditions.

To test a possible interaction effect of dosage and time, we used the same cluster statistics in a factorial design with dosage as a between-subjects factor. This resulted in a 2-by-2, mixed between-within-subjects two-way design.

We defined 4 cells: (12 mg, PRE), (12 mg, POST), (6 mg, PRE) and (6 mg, POST) where in each cell, the first factor is represented by ‘dosage’ and the second by ‘time’. In the first factor (between-subjects), we considered the two sub-groups of subjects (the 3 subjects with 12 mg perampanel and the 13 subjects with 6 mg). All the subjects participated in both PRE and POST conditions (within-subjects factor). Our analysis of the P60 pre- and post-perampanel was performed for each of the 4 conditions.

Then, we compared statistically the two effects: 12 mg_DIFF = (12 mg, POST)-(12 mg, PRE) and 6 mg_DIFF = (6 mg, POST)-(6 mg, PRE). Since two differences were compared, an interaction effect was tested. As our set-up is a mixed design, in this case we used an independent samples t-test for the cluster analysis.

#### TMS-induced EEG oscillations (TIOs)

For each TOI, pre-drug TIOs were statistically compared across the four DRUG conditions to assess their reproducibility. Moreover, for each DRUG condition and each TOI, post-drug vs. pre-drug statistical comparisons were performed to assess possible drug-induced changes in TIOs. Statistically significant differences were evaluated for each individual time–frequency region using channel-wise paired-sample *t*-tests. To control for multiple comparisons, the same cluster-based permutation approach^44^ was used, as described above for the TEPs.

#### PARAFAC

A permutation-based analysis was applied to test for significant differences pre- vs. post-drug on the four decomposed tensor components. The 5D tensor from which the original four tensor components were obtained, was randomly permuted in the elements of ‘subjects’ and ‘conditions’ for 1000 iterations. Next, each permuted 5D tensor was decomposed in four 1-rank tensors, as with the original dataset. Values of pre-drug measurement were subtracted from the post-drug values in the original and permuted tensor components to assess the effects due to drug intake in the TIOs. Then, a histogram of the ‘post–pre’ values obtained from the surrogate data was computed. The change in the original dataset was considered significant if its value outside the 2.5 percentiles of the ‘post–pre’ surrogate distribution.

## Results

TMS procedures were well tolerated by all subjects. In one case, a dosage of 12 mg perampanel caused dizziness, nausea and ataxia, which led to reduction of the dosage to 6 mg for the remaining 13 subjects (i.e., 3 of the reported subjects received 12 mg, the other 13 subjects received 6 mg of perampanel). Otherwise, drugs were well tolerated by all subjects, apart from slight nausea and/or dizziness reported after perampanel and dextromethorphan intake.

### Drug effects on RMT and MEP amplitude

The rmANOVA on RMT values revealed a significant DRUG × TIME interaction (F_3,45_ = 8.993, p < 0.001). Post hoc paired *t*-tests demonstrated an RMT increase (post-drug/pre-drug) after perampanel (mean ± SD, 1.09 ± 0.08; t_15_ = 4.11, p < 0.001) and nimodipine (1.04 ± 0.04; t_15_ = 2.91, p = 0.007), but not dextromethorphan (0.99 ± 0.07, t_15_ = 0.94, p = 0.36), compared with the non-significant RMT change under placebo of 0.97 ± 0.07.

Importantly, the rmANOVA did not reveal any significant effects of DRUG, TIME or DRUG × TIME interaction on MEP amplitude (Supplementary Table 3 in Supplementary Material). Thus, there were no changes in MEP amplitude that could have accounted for the drug effects on TEPs and TIOs as reported below.

### TMS-evoked EEG potentials (TEPs)

**Figure 1:**
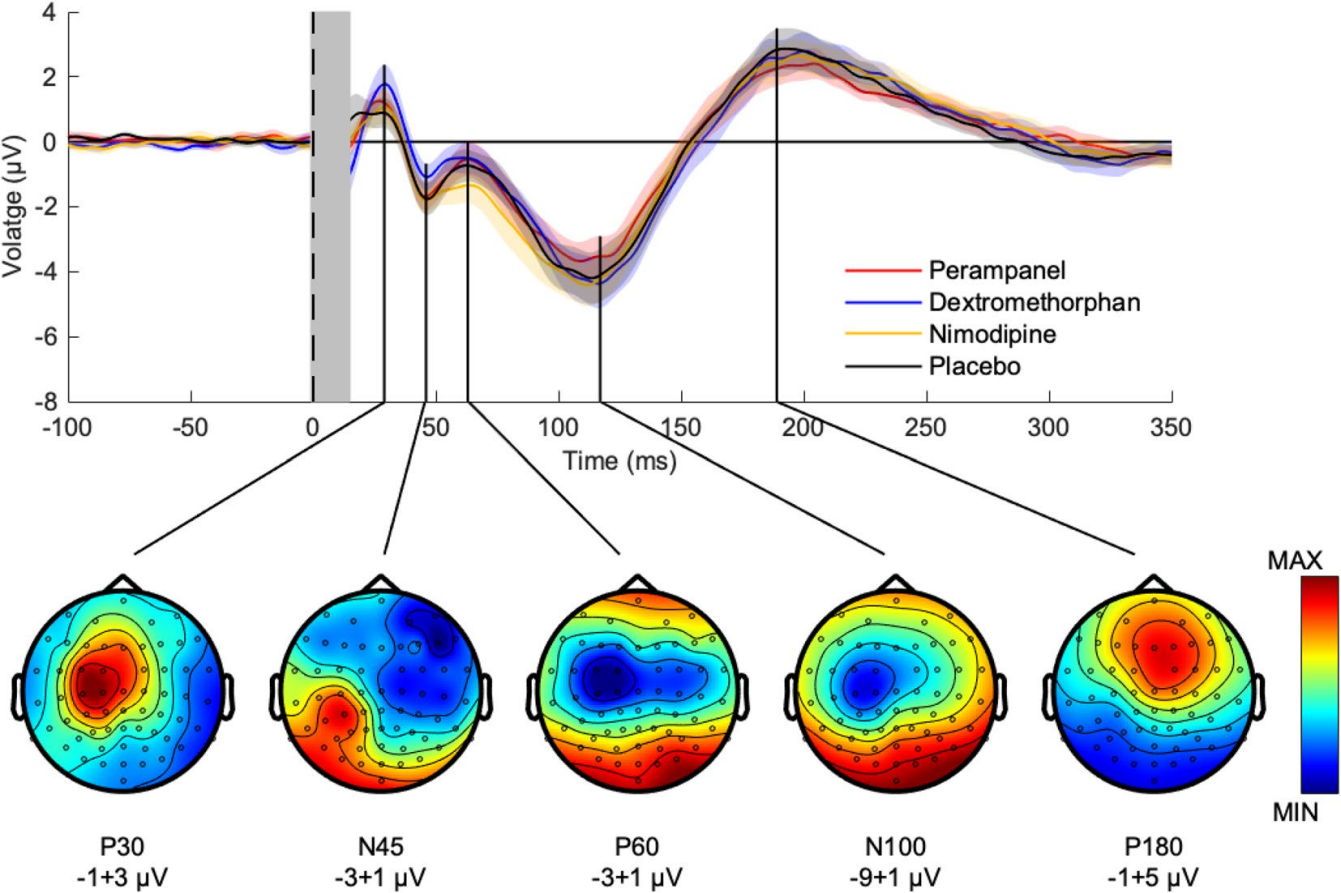
Group average of pre-drug TMS-evoked EEG potentials (TEPs) after stimulation of left motor cortex. Top panel: pre-drug TEPs averaged across all subjects (n = 16) and EEG electrodes for perampanel (red curve), dextromethorphan (blue curve), nimodipine (yellow curve) and placebo (black curve). Shades represent ± 1 SEM. The vertical gray bar represents the time window affected by the TMS artefact that was removed and interpolated. Bottom panel: pre-drug TEP topographies averaged across subjects (n = 16) and drug conditions. Each topography was obtained by averaging the signal in the respective TOI (P30: 16–34 ms, N45: 38–55 ms, P60: 56–82 ms, N100: 89–133 ms, P180: 173–262 ms). Data are voltages at sensor level (ranges indicated underneath the plots), while colors are normalized to maximum/minimum voltage. Figure generated with MATLAB R2016a (https://www.mathworks.com) and the Fieldtrip Matlab toolbox (http://www.fieldtriptoolbox.org).

Pre-drug TEPs and their topographical distributions (Fig. 1) were consistent with previous studies of single-pulse TMS over M1^12,14^. Pre-drug TEPs did not differ between the four DRUG conditions (all pairwise comparisons, p > 0.05).

**Figure 2:**
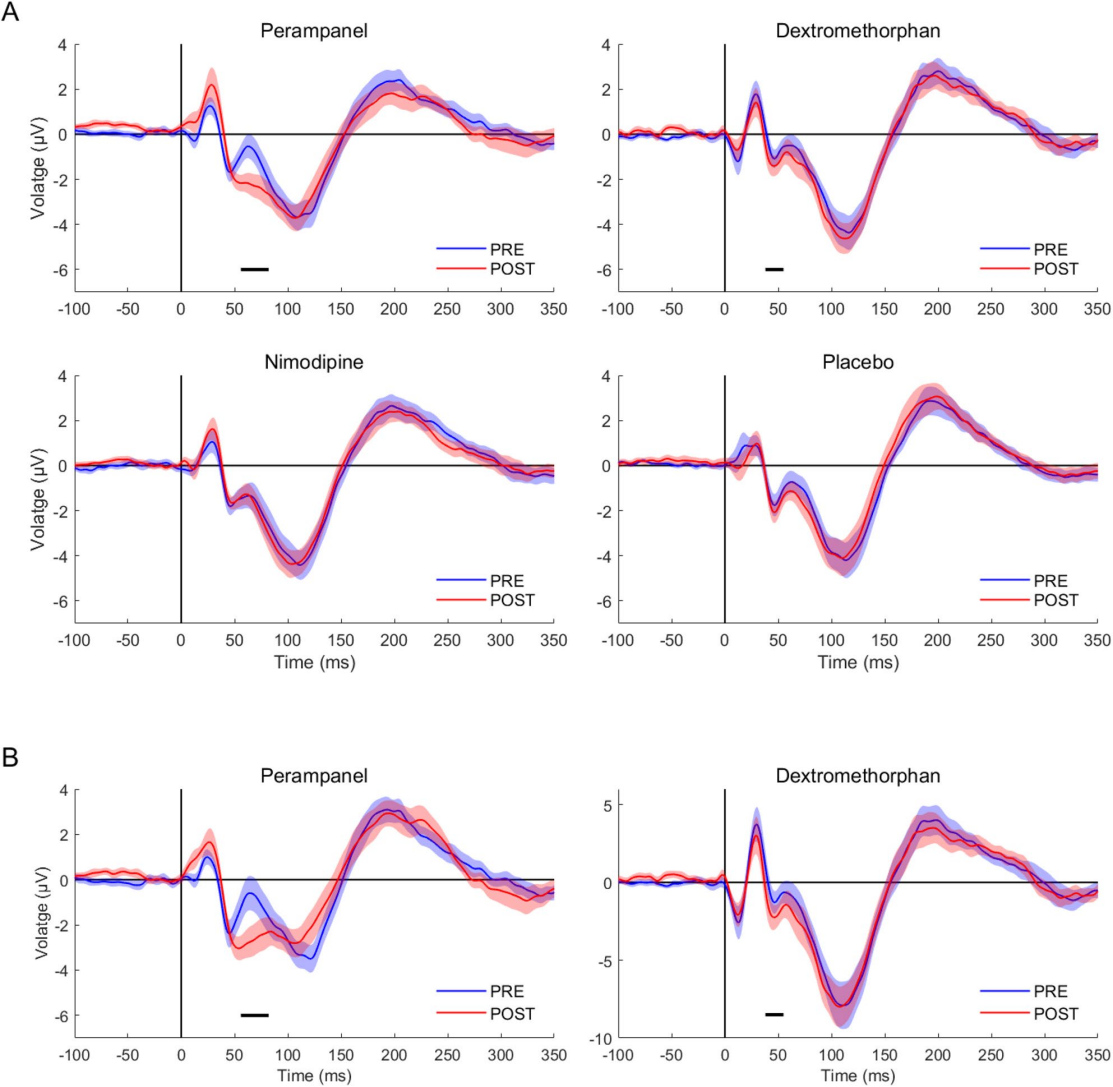
Group average of TMS-evoked EEG potentials (TEPs) pre- and post-drug intake. (**A**) Each panel shows the average TEP time course across subjects and all EEG channels of pre-drug (blue curve) vs. post-drug measurements (red curve) for the four drug conditions. Shades represent ± 1 SEM. Significant differences between the pre- and post-drug measurements are indicated with horizontal black bars. (**B**) To better elucidate the drug-induced changes of TEP components shown in (**A**), the same average TEP time courses are displayed for significant channels only (cf. Fig. 3). Shades represent ± 1 SEM. Figure generated with MATLAB R2016a (https://www.mathworks.com).

**Figure 3:**
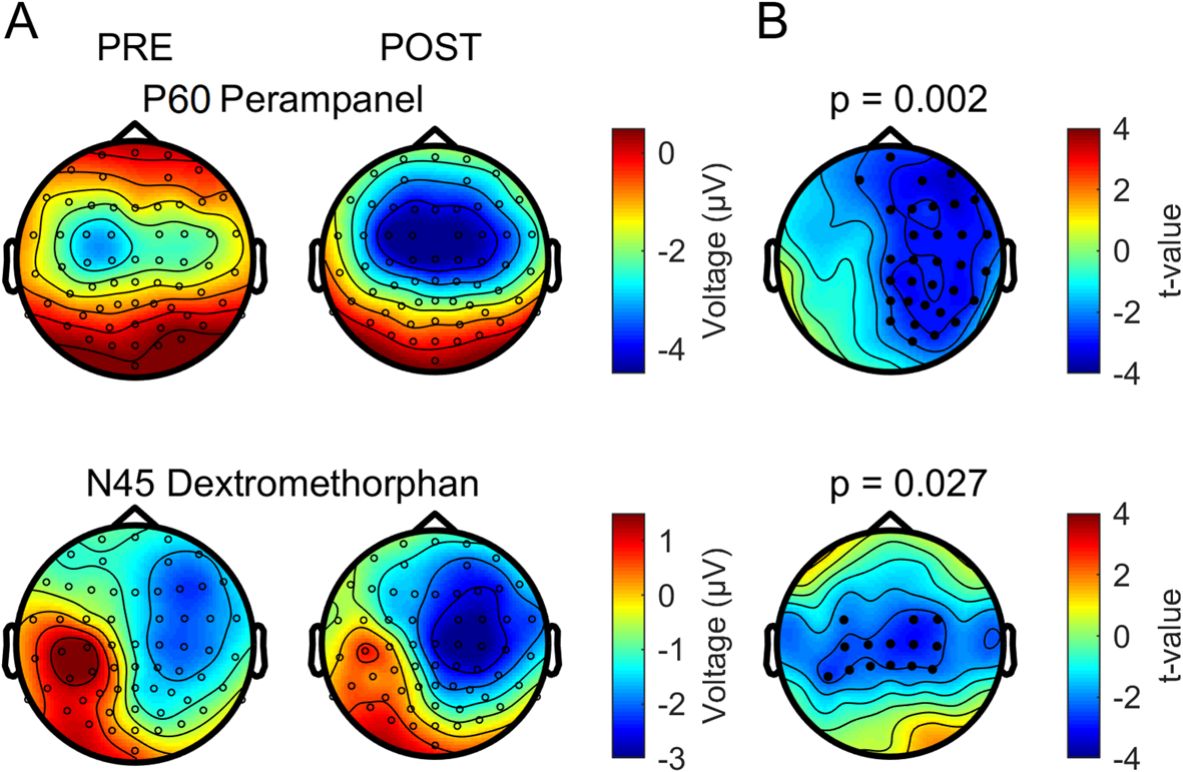
Topographical surface voltage maps for significantly different TMS-evoked EEG potential (TEP) components. (**A**) Topography of the P60 TEP before (left) and after (right) intake of perampanel (top row) and topography of the N45 TEP before (left) and after (right) intake of dextromethorphan (bottom row). (**B**) *T*-value statistical maps with channels belonging to significant electrode clusters (respective p-values are reported above the cluster topoplots) highlighted as black dots. Figure generated with MATLAB R2016a (https://www.mathworks.com) and the Fieldtrip Matlab toolbox (http://www.fieldtriptoolbox.org).

In the placebo and nimodipine conditions there was no significant difference in the post-drug vs. pre-drug measurement in any of the five TOIs (all p > 0.05; Fig. 2). Perampanel resulted in a decrease of the P60 amplitude (p = 0.002; Fig. 2A,B). This difference was expressed predominantly in EEG channels in the non-stimulated hemisphere (Fig. 3, top row). Dextromethorphan increased the N45 amplitude (p = 0.027; Fig. 2). The difference was expressed in a bilateral pericentral cluster of electrodes in the stimulated and non-stimulated hemisphere (Fig. 3, bottom row). Both the N45 dextromethorphan and the P60 perampanel cluster (Fig. 3) were still significant after applying the FDR correction.

Single subject data of drug-induced modulations of the P60 and N45 TEP amplitudes are displayed in Supplementary Fig. S2 (Supplementary Material) to illustrate consistency across subjects.

**Figure 4:**
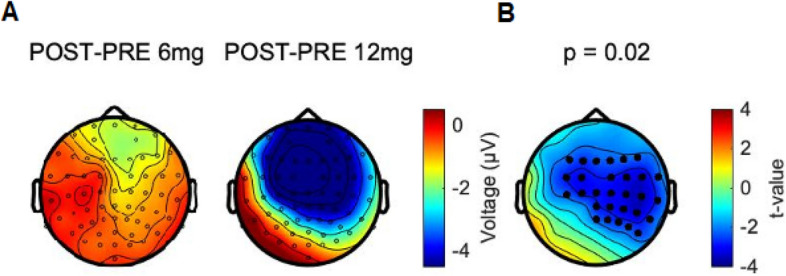
(**A**) Topographical maps of absolute changes (in µV) in power values (Post–Pre) in the P60 TEP under the two different dosages of perampanel (12 mg vs. 6 mg). (**B**) t-value topography of the difference of the two effects obtained with cluster-based statistics. Channels where a statistically significant difference was expressed between (Post–Pre 12 mg) and (Post–Pre 6 mg) P60 are indicated by black dots (p = 0.02). Figure generated with MATLAB R2016a (https://www.mathworks.com) and the Fieldtrip Matlab toolbox (http://www.fieldtriptoolbox.org).

The 2 × 2 interaction test on the dosage effects of TIME × DOSAGE of perampanel on the P60 revealed a marked effect due to the 12 mg compared to the 6 mg dosage in decreasing the P60 amplitude (Fig. 4A). The significant difference (p = 0.02; Fig. 4B) between dosage effects was detected in EEG channels in the stimulated and non-stimulated hemisphere, with a 66% overlap (21/32 channels) with the POST–PRE P60 cluster in Fig. 3B.

### TMS-induced EEG oscillations (TIOs)

**Figure 5:**
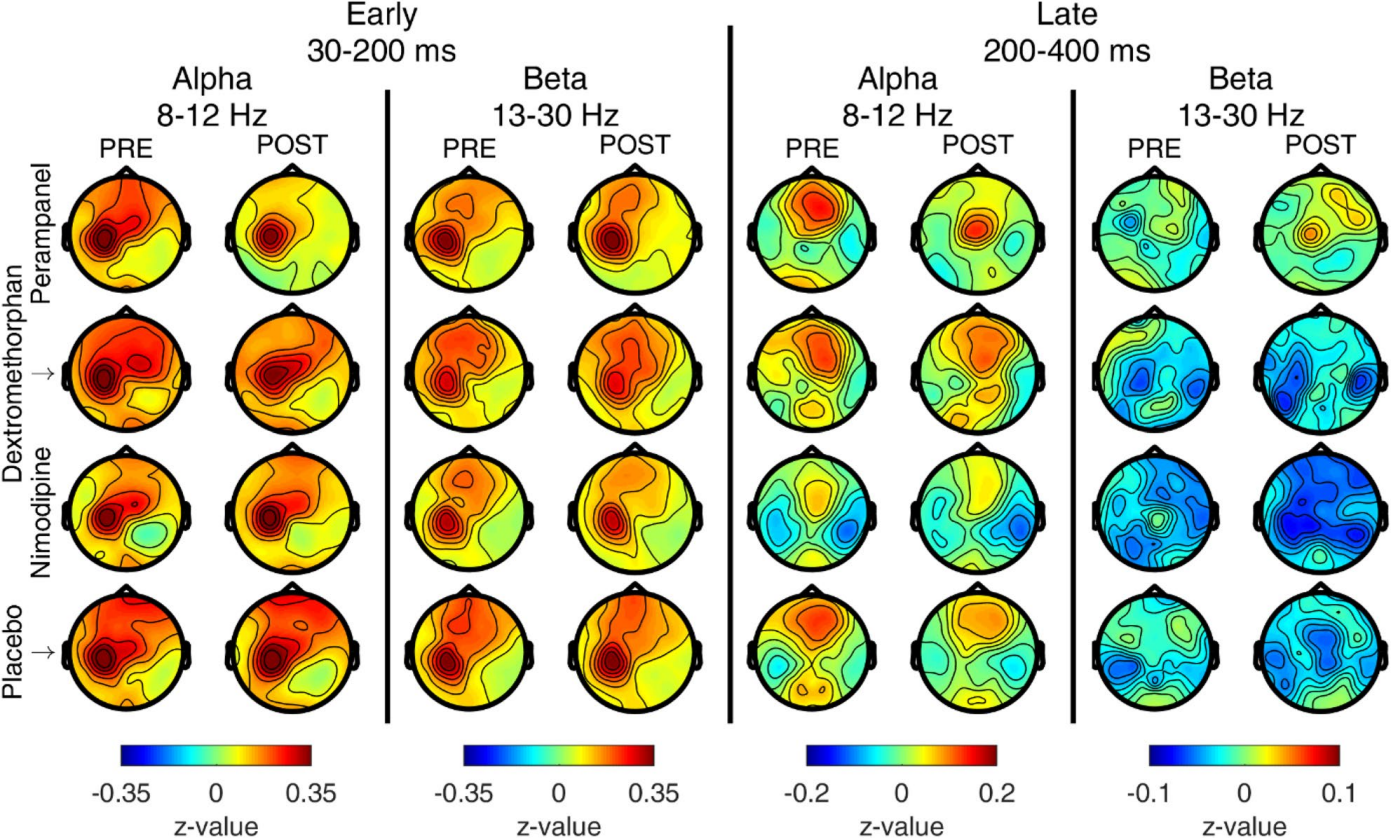
TMS-induced EEG oscillations (TIOs) pre- and post-drug application. In each panel, topographical maps of z-transformed baseline-normalized TIOs before (PRE, left columns) and after (POST, right columns) drug administration for all tested drugs (first row: perampanel, second row: dextromethorphan, third row: nimodipine, fourth row: placebo). The frequency bands of interest (alpha: 8–12 Hz, beta: 13–30 Hz) and the post-stimulus times of interest (early: 30–200 ms, late: 200–400 ms) are displayed in the topoplots as indicated at the top of this figure. Cluster statistics did not reveal any significant difference between post-drug vs. pre-drug TIOs for any of the tested drugs and time–frequency regions. Figure generated with MATLAB R2016a (https://www.mathworks.com) and the Fieldtrip Matlab toolbox (http://www.fieldtriptoolbox.org).

Pre-drug TIOs (Supplementary Fig. S3 in Supplementary Material) showed the typical early power increase followed by a late power decrease in the alpha and beta frequency bands^15^ and did not differ across DRUG conditions (all clusters p > 0.2). Comparison between post- and pre-drug induced oscillations did not result in any significant difference for any of the tested time–frequency regions of interest with the canonical data analysis (Fig. 5).

### PARAFAC tensor decomposition of TMS-induced EEG oscillations (TIOs)

**Figure 6:**
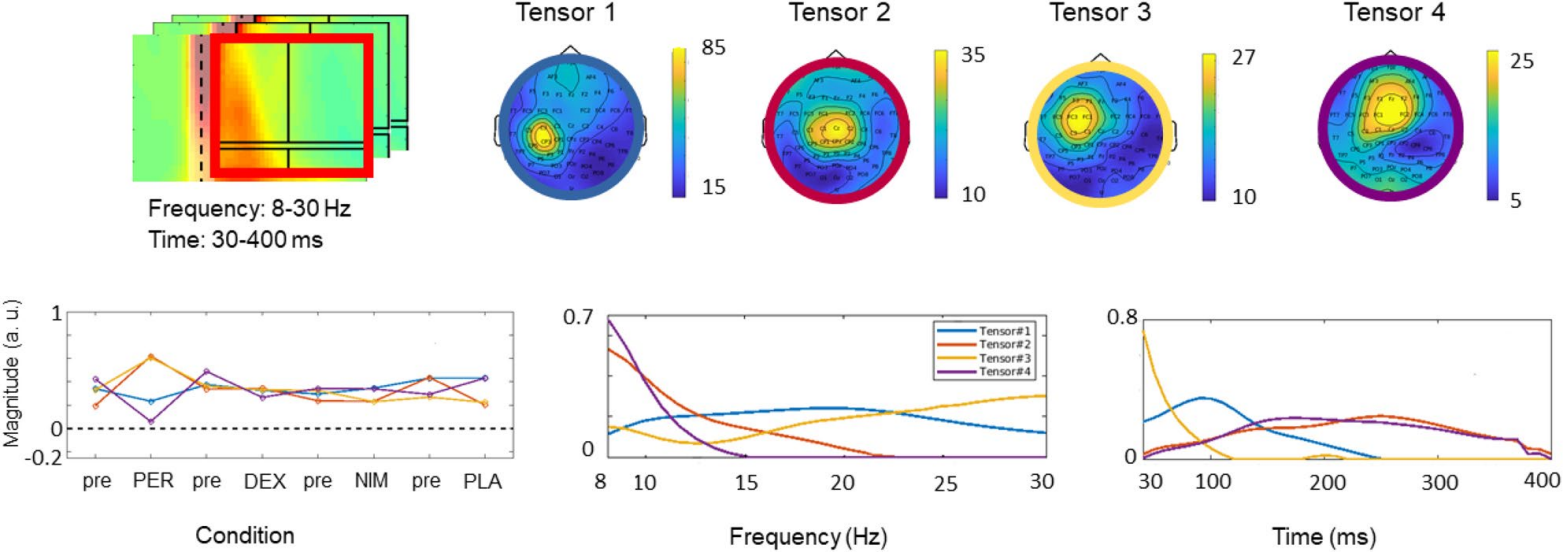
PARAFAC tensor decomposition of TMS-induced EEG oscillations (TIOs). Top row shows in the red square the time–frequency representation window employed for tensor decomposition analysis (the black lines denote the time–frequency intervals of the standard TIO analysis), and the topographies of the four tensor components (TCs) obtained from the induced data circled with four colors: blue, red, yellow and violet. The four TCs we obtained by extracting the most relevant components out of a 5D tensor with dimensions: Subject, Space, Drug Condition, Frequency, and Time. Bottom row, left plot: Drug-induced changes of the four tensors comparing pre- and post-drug data (PER, perampanel; DEX, dextromethorphan; NIM, nimodipine; PLA, placebo; the same colors as in top row indicate the different tensor components). Central plot: tensor frequency contents. Right plot: tensor time courses (time after the TMS pulse, in ms). Figure generated with MATLAB R2016a (https://www.mathworks.com) and the Fieldtrip Matlab toolbox (http://www.fieldtriptoolbox.org).

Figure 6 shows different dimensions of the four decomposed components, depicted in blue, red, yellow and violet, respectively. The first component (blue) had its peak in the C3 and CP3 electrodes (i.e., at and/or adjacent to the stimulated left sensorimotor cortex) and represented brain activity mainly in the beta range (peak at 19 Hz). On the time axis, this component had its maximum at ~ 100 ms after the TMS pulse, then it declined until zeroing at about 250 ms. The second component (red) involved the medial parietal area, peaking at the Cz electrode and characterized by low-frequency activity predominantly in the alpha range. It was active over the whole 400 ms, with a peak at around 250 ms. The third component (yellow) was centered on the FC3, C3 and FC1 electrodes, had a broad frequency spectrum, peaking at the limit of our range, at about 40 Hz, and showed a short time course with a maximum at 30 ms and zeroing at ~ 120 ms. The fourth (violet) component was located at frontal electrodes with a peak at Fz and consisted of low-alpha frequency content. It was active across the 400 ms with a peak at ~ 200 ms. Pre-post drug effects are reported in the left panel of the lower row of Fig. 6, showing an increase of the second (red) and third (yellow) tensor components after perampanel intake, while the other drugs had no effect.

An analysis on pre- vs. post-drug distribution values served to ascertain whether the strength value of the original measures in the single pre- and post-drug sessions were significantly different with respect to the distribution of strengths calculated from the shuffled datasets. This way, a sanity check on possible significant post- vs. pre-drug results due to preposterous significance of the original measures before drug intake was obtained. Significant components were found exclusively for the second and third tensor components *after* perampanel intake (p = 0.005 and p = 0.015, respectively). Importantly, pre-drug strength values were non-significant for each tensor component and all drugs (all p > 0.2). In the same way, all post-placebo tensor components were non-significant (all p > 0.2). Dextromethorphan and nimodipine also did not show significant components pre- or post-drug intake (all p > 0.1). This analysis was also meant to check for possible ‘spurious’ significant changes (i.e., significant differences obtained from non-significant pre- and post-drug original tensor strengths located at opposite sides *within* the non-significant, central part of the putative distribution).

**Figure 7:**
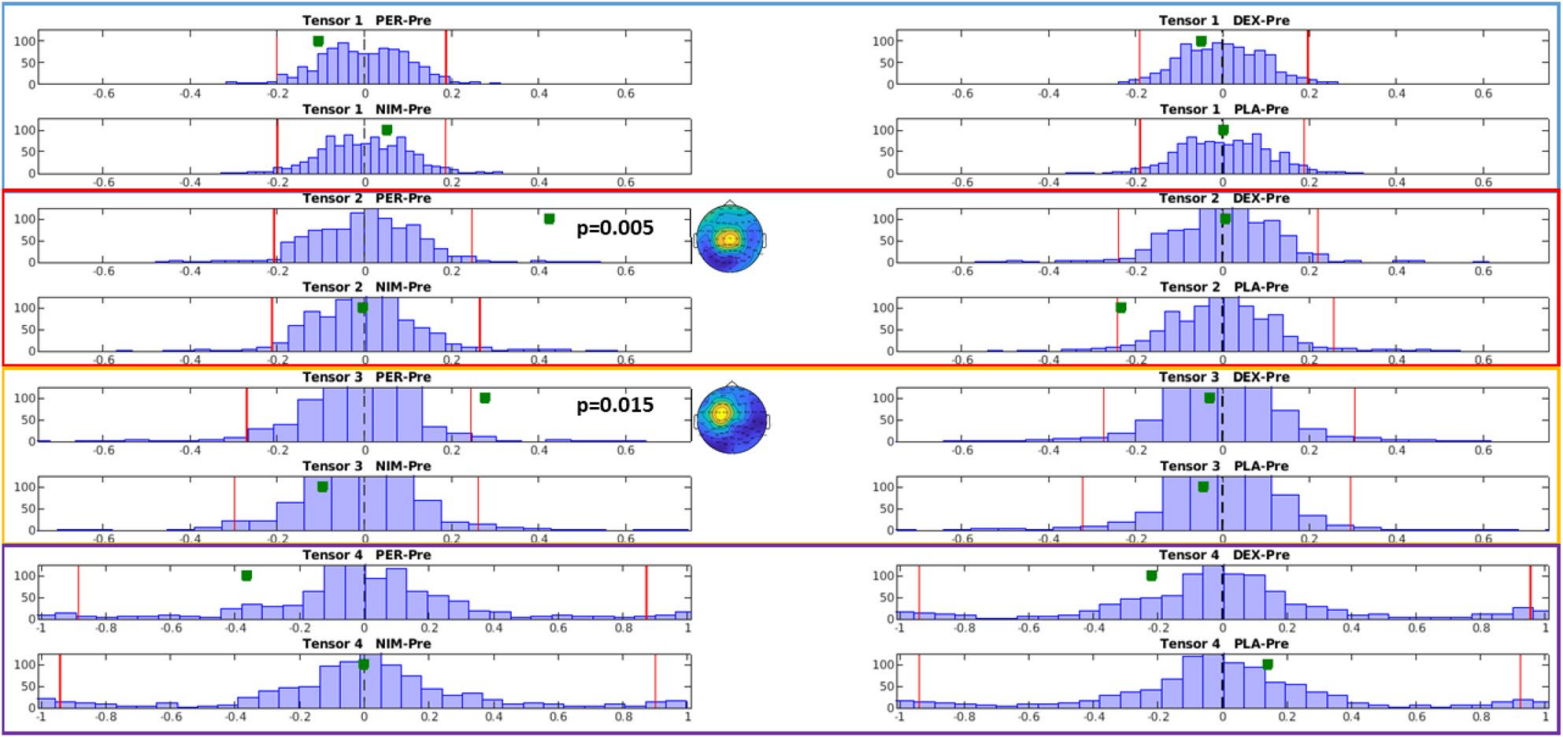
Strength differences of tensor components post–pre drug intake. The 16 histograms show the distribution of strength change post-drug vs. pre-drug out of 1000 iterations for the first (blue frame), second (red), third (yellow) and fourth (violet) tensor components. The shuffling of the data for creating the synthetic dataset was performed on the drug condition dimension. Within the histogram plots, the two vertical red lines define lowest and highest 2.5%. The green squares represent the post–pre difference in the original data (without shuffling). Significant increases were detected for the second and third tensor components after perampanel intake (p < 0.005 and p < 0.015, respectively). *PER* perampanel, *DEX* dextromethorphan, *NIM* nimodipine, *PLA* placebo, *Pre* pre-drug intake. Figure generated with MATLAB R2016a (https://www.mathworks.com).

Figure 7 shows histograms of strength differences post–pre drug intake. A significant increase of the second (red) and third (yellow) component was found due to perampanel intake (p = 0.005 and p = 0.015, respectively).

## Discussion

This study investigated the effects of anti-glutamatergic drugs on excitability of human cortex by TMS-EEG measures, in particular TEPs and TIOs, using different analytical approaches. The AMPA receptor antagonist perampanel decreased the amplitude of the P60 TEP component, while the competitive NMDA receptor antagonist dextromethorphan increased the amplitude of the N45 TEP component. The L-type VGCC blocker nimodipine and placebo had no effect on TEP amplitudes. While standard analysis of TIOs did not reveal significant drug effects, PARAFAC analysis showed that perampanel increased the activity of two data-decomposed tensors, one located in the stimulated sensorimotor cortex with high-beta frequency content, and one in the midline parietal area characterized by low-alpha frequencies. These differential effects are likely caused by differences in the specific modes of drug action as will be discussed in detail below.

### Increase of the amplitude of the N45 TEP component by dextromethorphan

**Figure 8:**
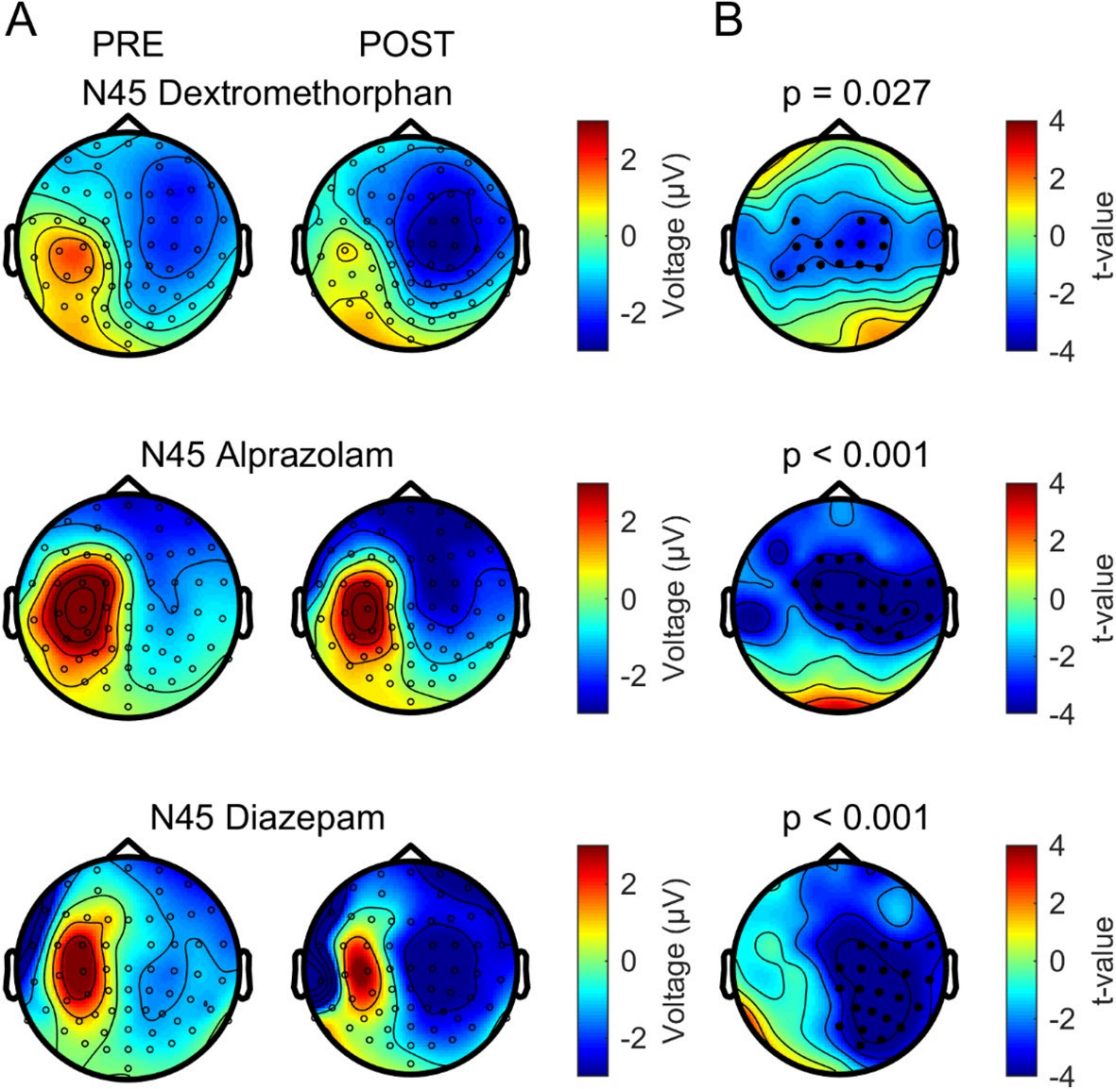
Comparison of the modulation of the N45 TEP by dextromethorphan and two classical benzodiazepines (alprazolam and diazepam, results adapted from Ref.^12^, with the consent of the authors). (**A**) Voltage surface maps of the N45 TEP recorded pre- and post-drug intake. (**B**) T-statistic maps of the N45 cluster post- vs. pre-drug differences. Electrodes of the significant clusters are denoted by black dots. Figure generated with MATLAB R2016a (https://www.mathworks.com) and the Fieldtrip Matlab toolbox (http://www.fieldtriptoolbox.org).

This study replicated lacking effects of dextromethorphan on RMT and MEP amplitude as consistently reported earlier^27,46^. At the level of TMS-EEG measurements, dextromethorphan showed a virtually identical effect as compared to benzodiazepines^12,13^ by increasing specifically the N45 TEP component (cf. Fig. 8). Therefore, the present data suggest that the N45 amplitude reflects excitation–inhibition balance of EPSPs and IPSPs (inhibitory postsynaptic potentials) evoked by the TMS pulse. This extends the view that the N45 amplitude specifically reflects GABAAergic inhibition^12–14^.

Of note, while the enhancing effects of the NMDA receptor antagonist dextromethorphan and benzodiazepines on the N45 amplitude are similar, dextromethorphan had no effect on the N100 amplitude in the non-stimulated hemisphere, while benzodiazepines decreased it^12,13^. Together, these findings support the idea that the N100 in the frontal cortex of the non-stimulated hemisphere reflects propagated neural activity controlled by GABAAergic but not glutamatergic neurotransmission.

### Decrease of the amplitude of the P60 TEP component by perampanel

The AMPA receptor antagonist perampanel increased the RMT. This finding corroborates the view that fast ionotropic glutamatergic neurotransmission through AMPA receptors at the synaptic connection of excitatory interneurons and pyramidal tract cells contributes to corticospinal excitability as tested by single-pulse TMS^47^.

The effect of perampanel on TEPs was specific to a reduction of the P60 amplitude. Of note, two of the three subjects who took the high 12 mg dosage showed the strongest decreases in P60 TEP amplitude (Supplementary Fig. S2 in Supplementary Material), suggesting a dose-dependent effect. Importantly, the reduction of P60 TEP amplitude was almost exclusively expressed in the non-stimulated right hemisphere (cf. Figs. 3 and 4), suggesting that the effect of perampanel is specific to interhemispherically propagated neural activity. This finding is in agreement with intrahemispheric and interhemispheric spread of epileptiform activity in rodent cortical slices that was not influenced by application of the NMDA receptor antagonist D-2-amino-5-phosphonovaleric acid (D-APV), but blocked by the AMPA receptor antagonist 6-cyano-7-nitroquinoxaline-2,3-dione (CNQX)^48,49^. The P60 TEP has not shown reactivity to any other of the so far tested central nervous system-active drugs^12–14^. The present findings suggest that the P60 TEP amplitude reflects glutamatergic (interhemispheric) signal propagation mediated by AMPA receptor activation. One recent study revealed a possible clinical relevance by demonstrating an exaggerated P60 response (which is probably equivalent to the P60 TEP in the present study) in a group of unmedicated patients with juvenile myoclonic epilepsy when compared to healthy controls and epilepsy patients on antiepileptic drugs^50^.

### Increase of TMS-induced EEG oscillations (TIOs) in the stimulated sensorimotor cortex and medial parietal areas by perampanel

The second and third PARAFAC tensor components with significant changes after perampanel intake were substantially complementary to each other concerning topography, frequency and time course (Fig. 6). The third tensor component can be considered as directly linked to the local effects of the stimulus because the signal under the FC3 and C3 electrodes covering the stimulated left sensorimotor cortex is involved in the tensor peak, its frequency content is predominantly in the high-beta band (Fig. 6), a characteristic oscillation response of the sensorimotor cortex to TMS^51^, and the component is expressed only in the first 100 ms, which is the typical duration of local oscillatory responses^51^. The increase of this tensor by perampanel is consistent with enhancing effects on beta-band power detected with resting-state magnetoencephalography (MEG)^52^ in healthy subjects, and in resting-state EEG of patients with epilepsy^53^.

In contrast, the second tensor component involved medial parietal channels with a time course lasting for the whole 400 ms of the analysis window, and low-alpha as main frequency content. Therefore, this component is probably linked to long-range intra- and interhemispheric signal propagation, which is typically mediated in the alpha-frequency range^42^. The finding of an increase of this tensor by perampanel is consistent with its increasing effect on alpha-band connectivity in the resting-state MEG, particularly within the parietal cortex^52^.

The maximum value in the topoplot of the non-significant first tensor component was 2.6 and 3.3 times stronger than the maximum of the second and third components, respectively. This first component appears to be directly linked to the stimulation, similarly to the third component, with a peak under the C3 electrode, and predominantly alpha and beta frequency content, but with a slower time course, peaking at 100 ms (Fig. 6). The first component did not show significant changes under any drug condition. Its large magnitude probably overshadowed the second and third tensor components in the canonical analysis of TIOs, and this may explain why no significant drug effects were revealed in that analysis (Fig. 5).

The fourth component remained without significant changes in any of the drug conditions. It was expressed mainly frontal, spanned over the full analysis window of 400 ms after the TMS pulse and had a low-alpha frequency content. It is probably related to long-range interhemispheric signal propagation^16^.

### Absence of nimodipine effects on TMS-evoked EEG potentials (TEPs) and TMS-induced EEG oscillations (TIOs)

L-VGCCs are expressed on dendrites of neurons throughout the central nervous system. They contribute to regulation of neuronal excitability. L-VGCCs open from their closed/resting state only upon strong postsynaptic depolarization^54^. In addition, L-VGCCs are not significantly involved in controlling glutamate release from presynaptic nerve terminals^28^. In line with this notion, nimodipine had no or only very minor effects on the MEP recruitment curve in previous studies^27^, and did not show any effect on TEPs and TIOs in the present study. A failure to obtain a nimodipine effect on TEPs or TIOs due to a too low dosage is unlikely, as the same single oral dose of 30 mg resulted in significant suppression of long-term potentiation and long-term depression-like plasticity in human motor cortex^27,29^.

Nimodipine increased RMT in the present study. This is in contrast to a previous study, which reported no change in RMT after intake of 30 mg of nimodipine^27^. The discrepancy is likely explained by subtle differences in the experimental protocols: RMT was measured 1 h after drug intake, i.e., during expected peak plasma time^55^ in this study, but rather 25 min after drug intake in the former study^27^.

### Study limitations

Some limitations of this study should be mentioned. First, due to the relatively limited sample size, it is possible that relevant findings were missed. The sample size was estimated on the basis of previous pharmaco-TMS-EEG studies, which demonstrated significant drug effects on TEPs and TIOs^12,15^. Therefore, it is unlikely that the sample size in the present study was underpowered. Second, perampanel dosage was reduced from 12 mg (first 3 subjects) to 6 mg (remaining 13 subjects). Given the result on the differential effect generated on the P60 tested by the 2-by-2 mixed design presented in Fig. 4 and the marked suppression of the P60 TEP amplitude in 2 of 3 subjects on 12 mg (Supplementary Fig. S2B in Supplementary Material), it is very likely that this effect would have been even more pronounced had all subjects taken the 12 mg dose. The decision on dose reduction during the ongoing study was driven by the adverse effects after 12 mg of perampanel. Importantly, the 6 mg dose of perampanel was still sufficient to produce significant effects on TEPs and TIOs, and the same single oral dose of 6 mg was used in other studies to demonstrate significant effects on MEG power and connectivity^52^. Third, it cannot be fully excluded that drug effects on TMS-evoked auditory and somatosensory potentials that contribute to the TEPs^56^ have played a role in the observed findings, as no sham stimulation has been applied for signal correction. However, this is unlikely, for two reasons: First, the peripherally evoked potentials are located in the midline^40,57^, while the TEPs modified by dextromethorphan (N45) and perampanel (P60) are lateralized potentials (Figs. 1, 3). Second, the peripherally evoked potentials are expressed no earlier than ~ 60–70 ms in the TMS-evoked EEG response^57^. Furthermore, dextromethorphan had no effect on the amplitude of early cortical somatosensory evoked potentials^58^, rendering a contribution of somatosensory evoked potential modulation by dextromethorphan to the observed increase in the N45 TEP amplitude highly unlikely. However, future pharmaco-TMS-EEG studies should corroborate this interpretation by adding a realistic sham condition to the experimental protocol that generates peripheral evoked potentials similar to real TMS but does not directly activate the brain^59^.

## Conclusions

Findings corroborate the general notion that TEPs and TIOs after single-pulse TMS of M1 can be used as markers of excitability and propagated neural activity in the human brain. Specifically, the effects of the NMDA receptor antagonist dextromethorphan extend our understanding of the N45 TEP amplitude to reflect excitation–inhibition balance regulated by NMDA and GABAA receptors. Furthermore, the suppressive effects of perampanel on the P60 TEP amplitude in the non-stimulated hemisphere and the increase of the midline parietal PARAFAC tensor component support the idea that this propagated activity is controlled by glutamatergic neurotransmission through AMPA receptors. The pharmacological characterization of TEPs and TIOs will be of utility in interpreting TMS-EEG abnormalities in neurological and psychiatric disorders with pathological neural excitability and/or signal propagation in brain networks.

## Supplementary Information

Supplementary Information.
