# Supplementary material for: TMS-EEG signatures of glutamatergic neurotransmission in human cortex
Source: Sci Rep. 2021 Apr 14;11:8159. doi: 10.1038/s41598-021-87533-z (PMC8047018; doi:10.1038/s41598-021-87533-z)
Supplement: Supplementary file 1 — Supplementary Information. [file 41598_2021_87533_MOESM1_ESM.docx]

**Supplementary Material**

**TMS-EEG signatures of glutamatergic neurotransmission in human cortex**

Paolo Belardinelli^1,2,3^, Franca König^1,2^, Chen Liang^1,2^, Isabella Premoli^4^, Debora Desideri^1,2^, Florian Müller-Dahlhaus^1,2,5^, Pedro Caldana Gordon^1,2^, Carl Zipser^1,2,6^, Christoph Zrenner^1,2^, Ulf Ziemann^1,2,*^

*^1^Department of Neurology & Stroke, University of Tübingen, Tübingen, Germany*

*^2^Hertie Institute for Clinical Brain Research, University of Tübingen, Tübingen, Germany*

[*^3^CIMeC, Center for Mind/Brain Sciences*](https://www.cimec.unitn.it/en)*, University of Trento, Italy*

*^4^Department of Basic and Clinical Neuroscience, Institute of Psychiatry, Psychology and Neuroscience, King's College London, UK*

*^5^Department of Psychiatry and Psychotherapy, Johannes Gutenberg University Medical Center Mainz, Germany*

*^6^Department of Neurology and Neurophysiology, University of Zurich, Balgrist University Hospital, Zürich, Switzerland*

*** Corresponding Author:** Prof. Ulf Ziemann, Department of Neurology & Stroke, and Hertie Institute for Clinical Brain Research, University of Tübingen, Hoppe-Seyler-Str. 3, 72076 Tübingen, Germany. Tel. +49 7071 2982049,

E-Mail: [ulf.ziemann@uni-tuebingen.de](mailto:ulf.ziemann@uni-tuebingen.de)

**
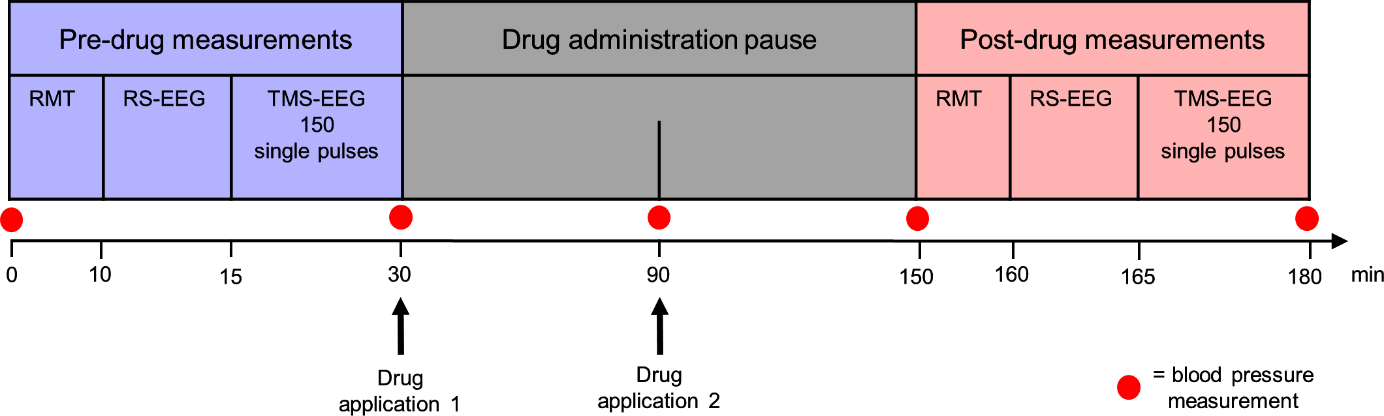
**

**Figure S1.** **Timeline of experiments.** Determination of resting motor threshold (RMT) at the beginning of pre- and post-drug measurements was followed by resting-state EEG (RS-EEG) and a block of 150 single TMS pulses over the left primary motor cortex with simultaneous EEG recordings (TMS-EEG). During the two-hour medication pause, drugs were administered at two time points (see Table 2 in Supplementary Material). One hour after the second drug administration, the post-drug measurements were performed in the same sequence as the pre-drug measurements. Blood pressure was monitored throughout the experiment (red dots). Figure generated with Microsoft Powerpoint (https://www.microsoft.com/en-ww/microsoft-365/powerpoint).

**
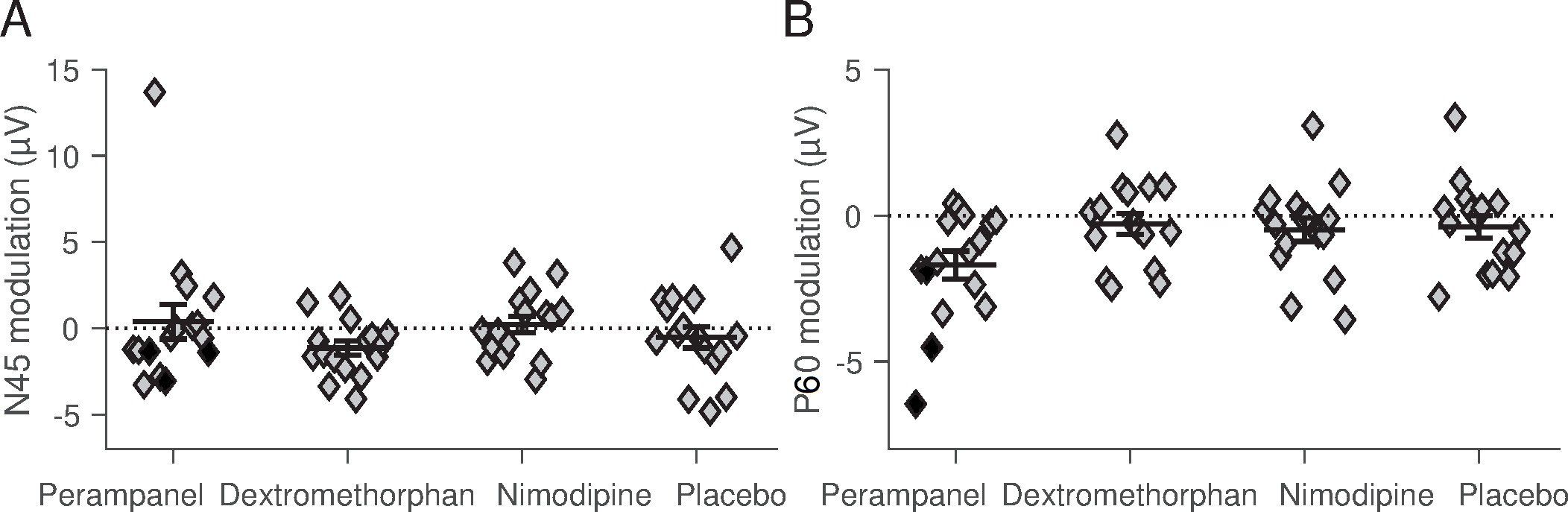
**

**Figure S2. Scatter plots of single subject drug-induced TMS-evoked EEG potential (TEP) changes**. TEP amplitude modulations (post-drug minus pre-drug) of the N45 (**A**) and P60 (**B**) TEP components for perampanel, dextromethorphan, nimodipine and placebo. For the investigated TEP components, amplitudes were calculated as the average voltage for identified significant channels for dextromethorphan (N45) and perampanel (P60) (cf. Fig. 3B). The changes in N45 and P60 TEP amplitudes of the three subjects who took 12 mg perampanel (in contrast to all other subject who took 6 mg) are marked in black. Error bars indicate mean ±1 SEM. Figure generated with MATLAB R2016a (<https://www.mathworks.com>).

**
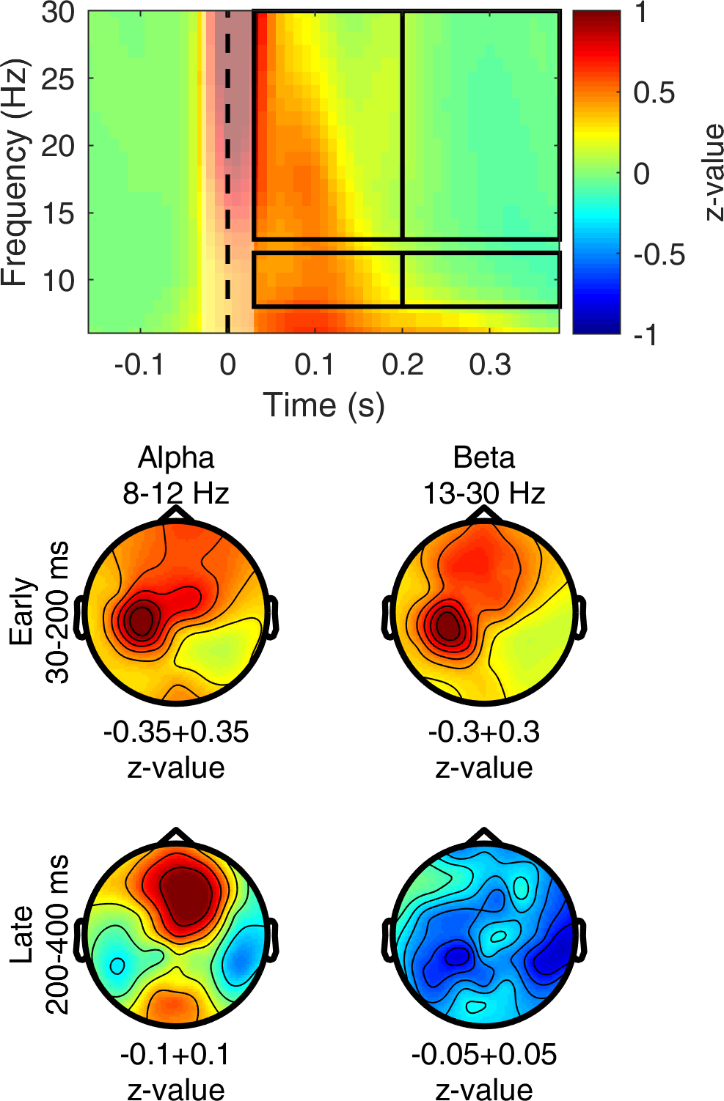
**

**Figure S3 – TMS-induced EEG oscillations (TIOs) before drug application.** Top: grand average across subjects (n = 16) and the four drug conditions of pre-drug TIOs at EEG channel C3, taken as representative of the activity of the stimulated left sensorimotor cortex. The squared absolute values of the TFR complex time series were trial-wise z-transformed based on the mean and standard deviation of the full-length trial and baseline-corrected. Black dashed line represents the time of the TMS pulse, the dull area surrounding it represents the time-frequency points possibly compromised by residuals of the TMS artefact and therefore excluded from the analysis. Black boxes enclose the four time-frequency regions of interest analyzed, in the alpha (8-12 Hz) and beta (13-30 Hz) frequency bands for the early (30–200 ms) and late (200-400 ms) time windows of interest. Bottom: Z-value topographical maps of the four time-frequency regions of interest. Figure generated with MATLAB R2016a (<https://www.mathworks.com>) and the Fieldtrip Matlab toolbox (http://www.fieldtriptoolbox.org).

**Supplementary Table 1**. Study drug single oral dosages, drug formulation, time of peak plasma concentration after intake (T_max_) and half-life time (T_1/2_)

| **Drug** | **Brand name** | **Dosage** | **Drug formulation** | **T_max_ [h]** | **T_1/2_ [h]** |
| --- | --- | --- | --- | --- | --- |
| **Perampanel** | Fycompa | 12 mg  (6 mg)^1^ | tablet | 1 [0.5-4]^2,3^ | 95^2^ |
| **Dextromethorphan** | Hustenstiller-ratiopharm Dextromethorphan | 120 mg | capsule | 1-2^4^ | 1.2-2.2 (CYP2D6-EM)  <45 (CYP2D6-PM)^4^ |
| **Nimodipine** | Nimodipin-HEXAL | 30 mg | tablet | 0.6-1.6^5^ | 1.1-1.7^5^ |
| **Placebo** | P-Tabletten Lichtenstein, 7, 8, 10 mm, Winthrop  Placebo Capsules UAT (University Pharmacy) | n/a | tablet  capsule | n/a | n/a |

^1^ Planned dose were 12 mg. Due to adverse effects, dose was reduced to 6 mg during the study. Three subject received 12 mg, the other 13 subjects received 6 mg.

^2^ Patsalos PN (2015). The clinical pharmacology profile of the new antiepileptic drug perampanel: A novel noncompetitive AMPA receptor antagonist. *Epilepsia* 56(1): 12-27.

^3^ Median [minimum-maximum]

^4^ Silvasti M, Karttunen P, Tukiainen H, Kokkonen P, Hanninen U, Nykanen S (1987). Pharmacokinetics of dextromethorphan and dextrorphan: a single dose comparison of three preparations in human volunteers. *Int J Clin Pharmacol Ther Toxicol* 25(9): 493-497.

^5^ PRODUCT INFORMATION NIMOTOP Nimodipine Bayer Resources.

Abbreviations: n/a, not applicable.

**Supplementary Table 2**. Timing of intake of study drugs and placebo relative to post-drug measurements (for more details, see Fig. S1 and main text).

| **Drug condition** | **Time point 1 (2 h prior to post-drug measurements** | **Time point 2 (1 h prior to post-drug measurements)** |
| --- | --- | --- |
| **Perampanel** | 4 capsules Placebo | 1 tablet Fycompa 12mg (6 mg) |
| **Dextromethorphan** | 4 capsules Hustenstiller-ratiopharm Dextromethorphan 30mg^1^ | 1 tablet Placebo |
| **Nimodipine** | 4 capsules Placebo | 1 tablet Nimodipin-HEXAL 30 mg |
| **Placebo** | 4 capsules Placebo | 1 tablet Placebo |

^1^ Dextromethorphan is only available as capsules, while perampanel and nimodipine are available as tablets. Dextromethorphan was administered 2 hrs prior to the post-drug measurements, due to its longer peak plasma concentration time compared to perampanel and nimodipine, which were given 1 hr prior to the post-drug measurements. This required a double-dummy design as indicated in this Table to secure blinding of participants and experimenters.

**Supplementary Table 3.** Pre-drug vs. post-drug measurements of resting motor threshold (RMT) and motor evoked potential (MEP) amplitudes (all data, mean ± SD). Significant changes post- vs. pre-drug are indicated in bold font.

| Drug | RMT pre-drug (%MSO) | RMT post-drug (%MSO) | MEP pre-drug  (mV) | MEP post-drug  (mV) |
| --- | --- | --- | --- | --- |
| Perampanel | 40.0 ± 6.4 | **43.6 ± 7.6** | 0.19 ± 0.31 | 0.16 ± 0.16 |
| Dextromethorphan | 40.9 ± 7.5 | 40.4 ± 6.7 | 0.17 ± 0.35 | 0.18 ± 0.20 |
| Nimodipine | 40.4 ± 7.2 | **41.8 ± 7.1** | 0.18 ± 0.07 | 0.19 ± 0.37 |
| Placebo | 40.8 ± 5.8 | 38.7 ± 8.3 | 0.18 ± 0.21 | 0.16 ± 0.16 |

Abbreviations: MSO%, percent maximum stimulator output.
